# The effects of operating height and the passage of time on the end-point performance of fine manipulative tasks that require high accuracy

**DOI:** 10.3389/fphys.2022.944866

**Published:** 2022-08-16

**Authors:** Ho Seon Choi, Hyunki In

**Affiliations:** Center for Healthcare Robotics, Artificial Intelligence and Robotics Institute, Korea Institute of Science and Technology, Seoul, South Korea

**Keywords:** end point accuracy, muscle fatigue, movement stability, proprioception, shoulder abduction

## Abstract

Sustained shoulder abduction, which results from an inappropriate worktable height or tool shape and long task hours, leads to an accumulation of muscle fatigue and subsequent work-related injuries in workers. It can be alleviated by controlling the table height or ergonomic tool design, but workers who are doing some types of work that require a discomfortable posture, such as minimally invasive surgery, cannot avoid these situations. Loads to the shoulder joint or muscles result in several problems, such as muscle fatigue, deterioration of proprioception or changing movement strategies of the central nervous system, and these are critical to work that requires a high accuracy of the upper extremities. Therefore, in this paper, we designed and conducted an experiment with human participants to discuss how an inappropriate height of the work-table affects the task performance of workers who are performing a fine manipulative task that requires high accuracy of the end point. We developed an apparatus that can control the height and has four touch screens to evaluate the end-point accuracy with two different heights. Eighteen adults (9 women and 9 men) participated in the experiments, and the electromyography of their shoulder muscles, their movement stability, and task performance were measured for the analysis. We found that inappropriate height of a table brings about muscle fatigue, and time elapsed for conducting tasks accelerated the phenomenon. Task performance deteriorated according to increased fatigue, and improved movement stability is not enough to compensate for these situations.

## 1 Introduction

Tasks that require repetitive manual movements for a long duration cause fatigue and illness in the upper limbs (Kilbom, 1994; Bernard and Putz-Anderson, 1997; Barbe and Barr, 2006). Fatigue reduces task performance, and if workers use sustained muscle loads to maintain performance at a certain level despite fatigue accumulation, it results in work-related musculoskeletal disorders due to inappropriate muscle use (Gallagher and Heberger, 2012; Barbe et al., 2013). Among the upper extremities, the shoulder joint is responsible for keeping the hand in the workspace during manual tasks, causing persistent static loads. And repetitive movements to be applied to the joint lead to disorders such as tendinitis (Barry and Enoka, 2007; Whittaker et al., 2019).

When performing a repetitive manual task, the shoulder fatigue is mostly generated due to the height of the worktable (Matern et al., 2001; van Veelen et al., 2002) and the misalignment between the work tool and the axis of the human joint (Matern, 2009; Perez-Duarte et al., 2013). Both factors impose abduction of the shoulder joint and add static loads to the shoulder muscles which accumulate fatigue in those muscles. And this leads to poor task performance and worker illnesses. Workers performing tasks at inappropriate table heights tend to have less time to reach a certain level of muscle fatigue and less accurate postwork manual movements than when progressing at ergonomic heights (Savole et al., 2002). High table heights require a larger workers’ shoulder abduction, and the longer it lasts, the shorter the workers’ time to reach fatigue (Chaffin, 1973; Kaur and Mishra, 2008). These fatigues increased the kinematic variability due to its dispersion of loads to other muscles, preventing the deterioration of stability, but workers gave up the performance of the task as a result (Gates and Dingwell, 2011). In contrast, tables at an optimal height alleviate these symptoms and allows workers to produce better task performance (Xiao et al., 2012).

If muscle fatigue develops in the upper limbs for some reason, it will also affect the task being performed. Prolonged time or increased intensity of exercise leads to increased oxygen consumption, and this results in increased production of lactic acid (Evans and Lambert, 2007). If this situation exceeds a certain level, the amount of production of lactic acid will increase more than the amount that is oxidized, and then the intracellular lactic acid will not be oxidized and accumulated in blood and muscles. This phenomenon causes muscle fatigue and affects performance of exercise or tasks. For example, the accuracy performance deteriorates by reducing the level of cocontraction when fatigue accumulates, even though the muscles have the ability to generate the same amount of force as in the prefatigue period. This is because the central nervous system (CNS) makes an optimal decision according to the criteria of metabolic efficiency. Therefore, in this situation, fatigued humans appear to have established a motor system strategy with a greater emphasis on energy economy than task performance.

Fatigue also deteriorates the proprioception of upper extremities (Blasier et al., 1995; Voight et al., 1996). Proprioception is afferent information related to joint speed, position, and limb and posture cognition, and it affects muscle sense as it is used for feedback elements for accurate motor control (Abd-Elfattah et al., 2015). So, if proprioception deteriorates, the accuracy of the end point can decrease. However, if it is not possible for subjects to give up end-point accuracy to perform tasks that require a precise process, which directly leads to the failure of the task, and the weight shifting of the CNS cannot also proceed. Therefore, a limitation for establishing a CNS motor strategy that deduces the optimal solution is generated, which causes different neuro-physical changes to be observed in workers.

End-point accuracy is also affected by muscle cocontraction. The mobility of the shoulder joint is achieved, among all, by the three heads of the deltoid (anterior, posterior, and medial) (Liu et al., 1997). When shoulder abduction occurs, the anterior and posterior deltoid contract and increase the joint stiffness and subsequent movement stability, while both are affected by fatigue and contribute to manual movement precision. As the stiffness of the joint increases, the destabilizing ability of impedance, which can compensate for the effect of external perturbation, is also enhanced, so the task can be performed while preserving the stability of the movement (Fu and Wang, 2009). Contrary to what is known that such stability can deteriorate if fatigue accumulates and proprioception decreases (Abd-Elfattah et al., 2015), Gates and Dingwell (2010) claimed that fatigue did not affect movement stability and that task performance (Movement tempo) was maintained. However, they placed an extra stage between tasks to produce fatigue. Cycle variation can be increased by increasing discomfort, and the characteristics of the task, such as monotonousness and repetitiveness, can adversely affect the shoulder (Hagberg et al., 1995; Luger et al., 2015). Therefore, it is not possible to observe the changes in the movement strategy corresponding to repetitive motion-induced muscle fatigue with their experiment. Therefore, analyzing the effect of fatigue caused by a task itself or the work environment on the movement stability and evaluating task performance of subjects who perform positioning tasks that require high accuracy would be helpful for understanding how the CNS manages those situations.

The purpose of this study is to learn how an improper work environment that causes shoulder muscle fatigue affects the movement state and task performance of workers performing tasks demanding a high-precision end point. We want to prove two hypotheses: 1) First one is that table of inappropriate height will adversely affect the performance of fine manipulative tasks. We thought that neuro-physiological elements related to performance of fine manipulative tasks will be affected by situations resulted from the inappropriate work environment. Because of task characteristics which require high end point accuracy, considerations of proprioception, cocontraction, and CNS strategy adjustment will be important, and these will be affected by the elapse of task time or table height. And these can result in subsequent adverse effects to task performance. 2) And second one is that there might be a relationship between movement stability of upper extremities and endpoint accuracy. Since the task performance usually improves when the system is stable, so we expected movement stability to serve as a link between physiological elements and end point accuracy. And we also considered other kinesiological elements like kinematic variability, joint stiffness, and muscle sense function. After recruiting participants to prove these hypotheses, we performed experiments with a repetitive high-precision positioning task at different heights and then analyzed the effects of muscle fatigue generated by the working environment on the movement and task performance of the subjects.

## 2 Materials and methods

### 2.1 Equipment (apparatus)

**FIGURE 1:**
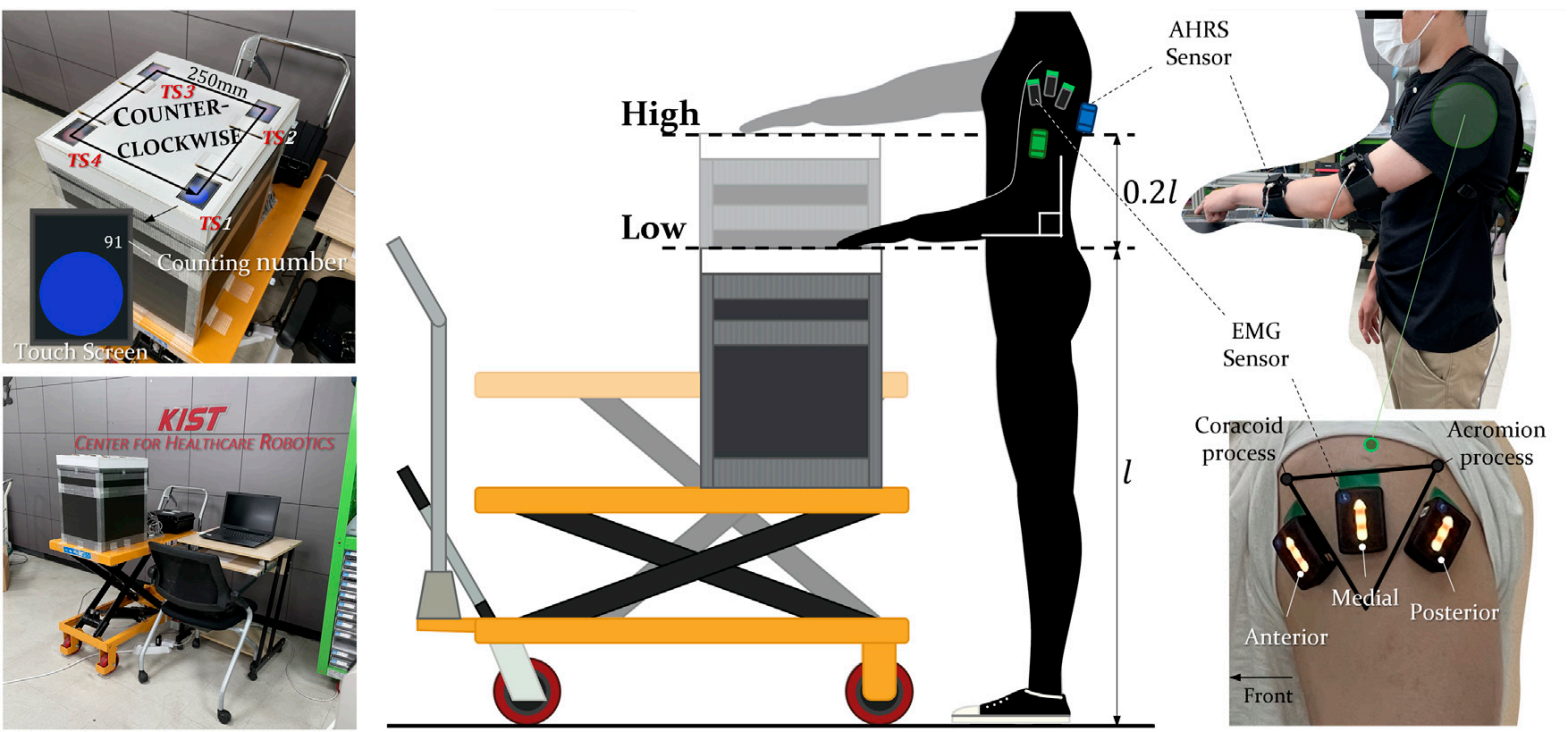
**(A)** Developed apparatus using resistive touch screen. The touch screen on the side closer to the body from the right is named TS1, and the number increases while turning counterclockwise. **(B)** Apparatus mounted on a height-adjustable shelf. **(C)** The ergonomics height of the elbow bent with 90 degrees of the Lower arm parallel to the floor is the LOW case, and the height equivalent to 120% of it is the HIGH case. **(D)** The AHRS sensors are attached to the lower, upper arm and the trunk to proceed with the experiment. **(E)** DELSYS Trigno sensors were attached to shoulder muscle to measure surface EMG signals.

Resistive touch screens mounted on a microcontroller board (STM32F429IDISCOVERY) developed by STMicroelectronics were used to develop a device capable of performing repetitive positioning tasks (Figure 1A). The touch screen was designed to display a circle with a diameter of 33.66 mm, and when subjects tapped the circle with the second finger, the cumulative number of trials up to that point was displayed and the color of the circle changed (Red↔Blue), which allowed participants to visually recognize tapping the touch screen. For repetitive tasks, a total of four touch screens were placed to form the vertices of a square with a side length of 250 mm, referring to a workspace for suturing or laparoscopic surgery (Lin et al., 2006; Leong et al., 2007; Azari et al., 2018). The touch screens were mounted on a height-adjustable shelf (Figure 1B), allowing participants to perform the repetitive task of tapping the four touch screens at different heights.

### 2.2 Protocols

First, three EMG sensors were attached to the deltoids of participants like Figure 1E and they also wore three AHRS sensors like Figure 1D. Then they tapped the touch screens in sequence from the lower right sensor (TS1 in Figure 1A) in a counterclockwise direction. And they were instructed how accurately they tapped the center of the circle when tapping the touch screen would be evaluated for the research. Pressing the four touch sensors in sequence was regarded as one cycle, and participants were instructed to conduct a total of 400 cycles each for two different heights. A case for each height, which was composed of 400 tapping cycles, was divided into 10 bins, and then one bin was composed of 40 trials. It was found through previous studies that the most suitable height of the worktable to perform precision work in the standing position is elbow height (Sanders and McCormick, 1998; Manasnayakorn et al., 2009). So, the two cases for experiment consisted of a height from the ground at the lower arm when the elbow was bent 90 degrees to the upper arm attached to the body, and a height equivalent to 120% of the former as shown in Figure 1C. In addition, participants visited the laboratory on different days for experiments with each case so that there was no need to consider the effects of fatigue between the two cases (at least 3 days apart).

### 2.3 Subjects

A total of 18 adults (28.3 ± 4.07 years/o), consisting of 9 females and 9 males, were recruited and participated in the experiment. The number of participants was determined based on a Monte Carlo model, which provides a probabilistic solution for random sampling (Bates et al., 1992; Dufek et al., 1995). According to the model, when conducting an experiment to compare a difference of 1 standard deviation unit with 20 participants, if number of trials is one or three, the statistical powers are 72.4 and >90%, respectively. Additionally, if the number of participants is 10 and the standard deviation unit is 0.5 with 15 or more trials, the statistical power will exceed 90%. Our experiments had 18 participants, and the size of the group used for comparison at different heights was composed of 400 cycles and 10 bins (which included 40 cycles) for the comparison in time elapsed so that we could secure enough statistical validity, although we did not calculate the exact validity value. All participants were healthy people who had never experienced any illness or disability in their arms or motor nerves. All experimental procedures were approved by the Institutional Review Board of Korea Institute of Science and Technology (2021–032). Participants were recruited based on voluntary intent through documents for recruitment, and after verbally explaining the contents of the experiment, they signed consent forms before starting the experiment.

### 2.4 Measurements

#### 2.4.1 Electromyography and muscle fatigue

A total of three surface EMG sensors (TrignoTM Wireless Biofeedback System developed by DELSYS) were used and attached to the anterior, medial, and posterior deltoids (Figure 1E). Using an eye-brow pencil that is harmless to the human skin, a triangle with the acromion process and coracoid process as vertices was drawn for dividing the position of the three muscles and was used to identify the position of the EMG sensor in the experiment for different days.

Each sensor measured the EMG signal at a rate of 1926 Hz, and postprocessing of the measured data was performed using DELSYS′ EMGworks Analysis program. A Butterworth bandpass filter with a cutoff frequency of 10–950 Hz was applied for noise reduction (Konrad, 2005). Then, a window length of 0.125 s and an overlap of 0.065 s (50%) were applied to calculate the root mean square (RMS) value to understand muscle activity (Lee et al., 1994; Menon et al., 2017; Tepe and Demir, 2020). Before the experiment started, a maximum voluntary contraction (MVC) for deltoids was conducted, and RMS values were calculated as ratios to this.

The median frequency (MF) was also calculated using a window length of 0.125 s and an overlap of 0.0625 s to determine the level of fatigue. The frequency that divides the power spectrum density of a surface EMG into two identical areas is called the MF (Brody et al., 1991). According to the studies using this method, it was found that as the muscle fatigue accumulated, the high frequency band decreases and the low frequency band increases, so that the frequency spectrum shifts to the low frequency band (Moxham et al., 1982; De Luca, 1984). Therefore, MF usually tends to decrease as fatigue accumulates (Cifrek et al., 2009), and we also measured muscle fatigue using this method.

The MF data were calculated by dividing the 400 cycles into 10 bins (corresponding to 40 cycles) and converted into a ratio to the value for the first bin, allowing comparison between different experiments. Furthermore, the average values of the 10 bins were calculated as a representative value of one case and used for comparison by height.

#### 2.4.2 Muscle cocontraction

In general, a cocontraction index (CCI), which represents the degree of cocontraction, was calculated as follows (Missenard et al., 2008; Cowley and Gates, 2017):

$$\mathrm{CCI}=\frac{\int \mathrm{EM}G_{\min}\mathrm{dt}}{\int \mathrm{EM}G_{\mathrm{AGO}}+\mathrm{EM}G_{\mathrm{ANT}}\mathrm{dt}}\times 100$$

where $EMG_{AGO}$ and $EMG_{ANT}$ are the RMS values of agonist and antagonist muscles, respectively, and $EMG_{min}$ represents the lower value between $EMG_{AGO}$ and $EMG_{ANT}$ at each point. EMG signals simultaneously generated by the agonist and antagonist are used to increase the joint stiffness, so the CCI is used to represent those characteristics. Of the three EMG sensors used, the CCI was calculated using the RMS of the two EMG sensors attached to the anterior and posterior deltoids, which were converted to a ratio to the MVC, excluding the medial deltoid sensors. The CCI was calculated separately for each stage (ST), and ST specifies the section between each touch screen, which consisted of a total of four. ST1 was the interval between TS1 and TS2, after which the number increased. In addition, the CCI was calculated for all stages tapping in a cycle from TS1 and returning to it.

#### 2.4.3 Movement variability and local dynamic stability

Since a human arm is a system of multiple degrees of freedom, the CNS can establish various motor strategies for any given redundant manual task. Therefore, even if the same task is executed repeatedly, variation will occur in every cycle. In addition, these variations are affected by the fatigue of the muscles used to perform the task (Kang and Dingwell, 2009; Gates and Dingwell, 2011; Longo et al., 2018a), so it should be measured to evaluate how height-induced shoulder muscle fatigue affects arm movement. For this, AHRS sensors (EBIMU-9DOFV5) developed by E2BOX were used, which measures 3-axis acceleration with gravity removed. A total of three sensors were used and worn with straps and vests on the lower arm, upper arm, and trunk (Figure 1D). Acceleration data were divided with the first touch screen signal so that each cycle had one profile. After that, the mean of the standard deviation of 40 cycles was calculated and converted into a kinematic variability (KV) value representing one bin (Longo et al., 2018b). And the Euclidean norm of three axis variabilities was calculated for each sensor so that a total of 3 KVs were used ( $KV_{LA}$ , $KV_{UA}$ , and $KV_{TR}$ representing the lower, upper arm, and trunk, respectively).

Local dynamic stability is a method for the quantification of movement stability using the largest Lyapunov exponent (LLE), which represents how the initial perturbation at a certain point affects the distance between the nearest neighbors and how much the two trajectories diverge over time during a repetitive motion (Abarbanel, 2012). It has usually been applied to gait stability but has recently been used for dynamic or positioning tasks of arm movement (Kellis et al., 2003; Longo et al., 2018a). Individual variables are required to calculate the LLE, but if we only measure and use the motion-related values of one segment, it will show partial stability, and it will be difficult to proceed with the analysis of the overall postural stability. To solve these problems, a method is used to measure the variables of all the segments to be evaluated and apply principal component analysis to them. All the measured values are decomposed into several principal components that represent the complete movements, and the LLE can be calculated with those values to evaluate the movement stability (Daffertshofer et al., 2004). Longo et al. (2018b) constructed the state space for local dynamic stability using only the first principal component of the measured values representing motion. However, we wanted to include more principal components and increase the degree to which they represent the entire data. Therefore, according to these methods, we extracted three dominant principal components from nine acceleration values measured by the lower arm, upper arm, and trunk, which can represent the complete motions of the upper extremities. The calculated LLE from these values was used to represent the movement stability of each bin. For the calculation of the LLE, cycles were normalized and interpolated with 100 data. For evaluating fall risk in gait analysis, 0.5–1 cycle was used for calculating the range for the LLE, but we do not know the optimal magnitude of the range for arm movement, so we calculated LLEs with 5%, 10%, quarter-, and half cycles for ranges, and the LLEs for each case are $\lambda_{5}$ , $\lambda_{10}$ , $\lambda_{Q}$ , and $\lambda_{H}$ , respectively.

#### 2.4.4 Movement accuracy

Movement accuracy was measured to evaluate the performance of the positioning action of the fine manipulative task. The size of the touch screen was 2.4 inches, and it had a 320 × 240 resolution so that one pixel had a size of 0.153 mm. The diameter of the circle on the touch screen was 16.83 mm (110 pixels). Participants were instructed to tap the center of the circle as accurately as possible, and the movement accuracy was evaluated to determine how the tapping point deviated from the center of the circle. The deviation between the center of the circle and the distance between the radius and the tapping point were transformed to ratios and used for evaluating the accuracy score (AS):

$$\mathrm{AS}=\frac{r_{\mathrm{circle}}-d_{\mathrm{tapping}}}{r_{\mathrm{circle}}}\times 100$$

where $r_{circle}$ is the radius of the circle and $d_{tapping}$ is the distance between the tapping point and the center of the circle.

#### 2.4.5 Task time

Task time for each cycle was measured by a microcontroller board (STM32F407DISCOVERY) based on the tapping timing of the first touch screen, and the mean value was calculated with 40 cycles representing one bin.

#### 2.4.6 Data acquisition

An STM32F429DISCOVERY measured the location of the tapping signal on the touch screen with 1,000 Hz data frequency, and the signal data for the four touch screens were transferred to the STM32F407DISCOVERY through a controller area network. The STM32F407DISCOVERY also operated with a 1,000 Hz timer, and it saved the received tapping signals and calculated the task time based on those. Three AHRS sensors (EBIMU-9DOFV5) sent 3-axis acceleration data to the STM32F407DISCOVERY through integrated circuit communication for saving. A TrignoTM Wireless Biofeedback System generated a trigger signal when the data logging started, and STMStudio, which is the software for data logging of microcontroller boards developed by STMicroelectronics, has a function for automatically starting data logging with a trigger signal. These were used to align the time domains of all measured data.

### 2.5 Predictive simulation with OpenSim

Conclusions for the movement strategy of the CNS can be less reliable when they are only deduced from the results of clinical experiments with human subjects. For example, the opinion that the CNS places more weight on metabolic efficiency when fatigue accumulates in the body is deduced from clinical experiments. However, if the results of a simulation with a musculoskeletal model in a computer are added, the conclusion can be supported, which can be more reliable. OpenSim is an open-source software program for dynamic simulation of movement (Delp et al., 2007), and we can obtain the information for the movement strategy of the musculoskeletal model corresponding to the weight ratio of the task goals. Although it cannot be sure that the musculoskeletal model is the same as that of the human body and the optimization tool is not exactly the same as the CNS operation principle, these are helpful for enhancing the reliability of the conclusion for discussion with the experimental results.

#### 2.5.1 Musculoskeletal model

**FIGURE 2:**
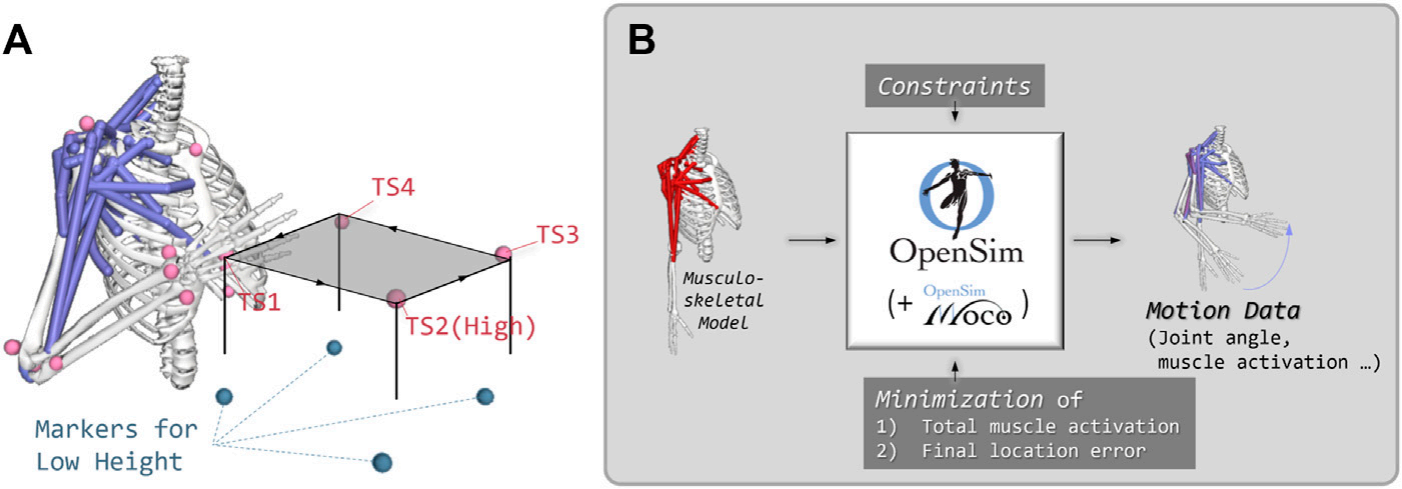
**(A)** Setup for predictive simulation with OpenSim and TS means touch screen. **(B)** Process of predictive simulation based on OpenSim MOCO toolbox.

The upper limb model of Fox et al. (2021) was used for simulations (Figure 2A), and it is well explained in their METHODS section. However, this model only has muscles related to shoulder joint movements, so we added three additional muscles for the elbow joint: the triceps brachii muscle as the extensor and the long and short heads of the biceps brachii muscle as the flexor. These three muscles have the same parameters as the others (Millard and Delp, 2012). In the case of the triceps brachii muscles, a wrap cylinder was used to instruct the path of the muscle. Every piece of information about the additional muscles, such as locations, paths, and maximum isometric forces, is included in the STO file attached in the Supplementary Material.

#### 2.5.2 Predictive simulations with OpenSim MOCO

OpenSim MOCO is a toolbox for predictive simulation using the musculoskeletal model of OpenSim (Dembia et al., 2020), and it uses direct collocation as an optimization method. Direct collocation is somewhat complex for beginners, but OpenSim MOCO created a toolbox for easy use. This toolbox is linked with OpenSim, and if we set the musculoskeletal model to be optimized with the objective functions or goals and give them each weight, then it calculated the optimized movements during a certain time with direct collocation methods, as shown in Figure 2B. Goals for optimization were the minimization of the location error between the final location of the movement and the designated location or the minimization of the muscle activation of all attached muscles for entire movements. The Python scripts used for predictive simulation with OpenSim MOCO are also attached in the Supplementary Material.

We attached a marker to the hand segment to minimize the final location error. We also located markers at the positions of the touch screens for the two different heights, so there were eight markers, as shown in Figure 2A. First, simulation of pointing to each touch screen marker from a neutral position while standing at attention was performed. The weights were 20 for both minimization of the muscle activation and final location error of the end point. We extracted the calculations of the model for each case, and these were used for the initial posture in simulations for pointing to the next touch screen. Predictive simulations were conducted for each stage (mentioned in Section 2.4.2), and we changed the weights for minimization of the total muscle activation. The weight for minimization of final location error was fixed at 20, and the weight for muscle activation was changed to 0.1, 0.5, 1, 2, 3, and 4.

### 2.6 Statistical analysis

We divided the 400 cycles into 10 bins and then calculated the mean and standard deviation for each bin. After that, for intercase comparison, those values were transformed to ratios (0–1) with regard to those of the first bin. In the case of the results with higher table heights, they were transformed to ratios with regard to the first bin of the results of the lower table heights except the case of the median frequency. The results of median frequency were transformed ratios regarding the first bin of each height. Then, the normalized data were grouped into one group regardless of the participant, and the number of data points, which equals 18 (number of participants) × 10 (number of all bins), were included in the group corresponding to one height. Paired t-tests were then performed between the two groups to observe height differences (*p* < 0.05). To observe changes over time, 10 bins were converted to five periods, and one period included 18 (number of participants) × 2 (number of included bins) sets of integral data. One-way repeated measures ANOVAs were performed for each height separately to determine whether there were any periods with significant differences. And if it appears that there is a significant difference between the groups for a certain height based on the results of ANOVA, then we did the paired *t*-test for 4 times to find significant differences compared to first period (for observing the change over time). And Bonferroni correction was used for correcting the significant level (*p* < 0.0125) to minimize the effect of the error due to multiple comparison.

## 3 Results

### 3.1 Electromyography

**FIGURE 3:**
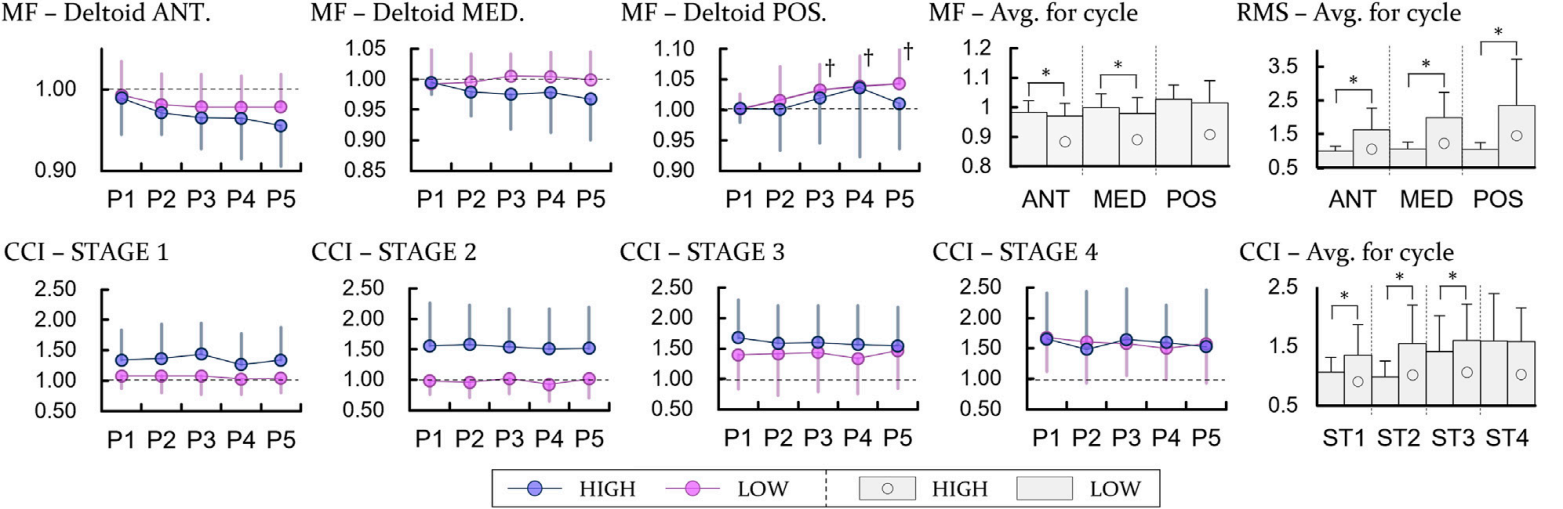
Experimental results related to the EMG. P and ST indicate period and stage. The units of all variables except MF are ratio to the result for the first bin of the LOW height. The unit for MF is ratio to the results for the first bin of each height. ANT, MED, and POS specify anterior, medial, and posterior deltoid, respectively. † represents a significant difference with regard to P1 (*p* < 0.0125). For comparing between the height, * means a significant difference with *p* < 0.05.

Significant fatigue was not observed on all shoulder muscles measured when performing the tasks. In the case of the anterior and medial deltoid, the median frequency tended to decrease, but it was not significant. However, in the height comparison, the MFs of the anterior and medial deltoids in the experiment with the HIGH heights were lower than those with the LOW heights (*p* < 0.05), and we determined that the manipulated circumstances added fatigue to the shoulder muscles as intended (Figure 3).

It was shown that the RMS values of the EMG increased as the height increased for all three measured muscles (*p* < 0.05). No change over time was observed in the RMS values and CCI results. However, in the case of the CCI, increases at ST1, ST2, and ST3 in the HIGH heights were discovered with significant differences (*p* < 0.05).

### 3.2 Cycle variation

**FIGURE 4:**
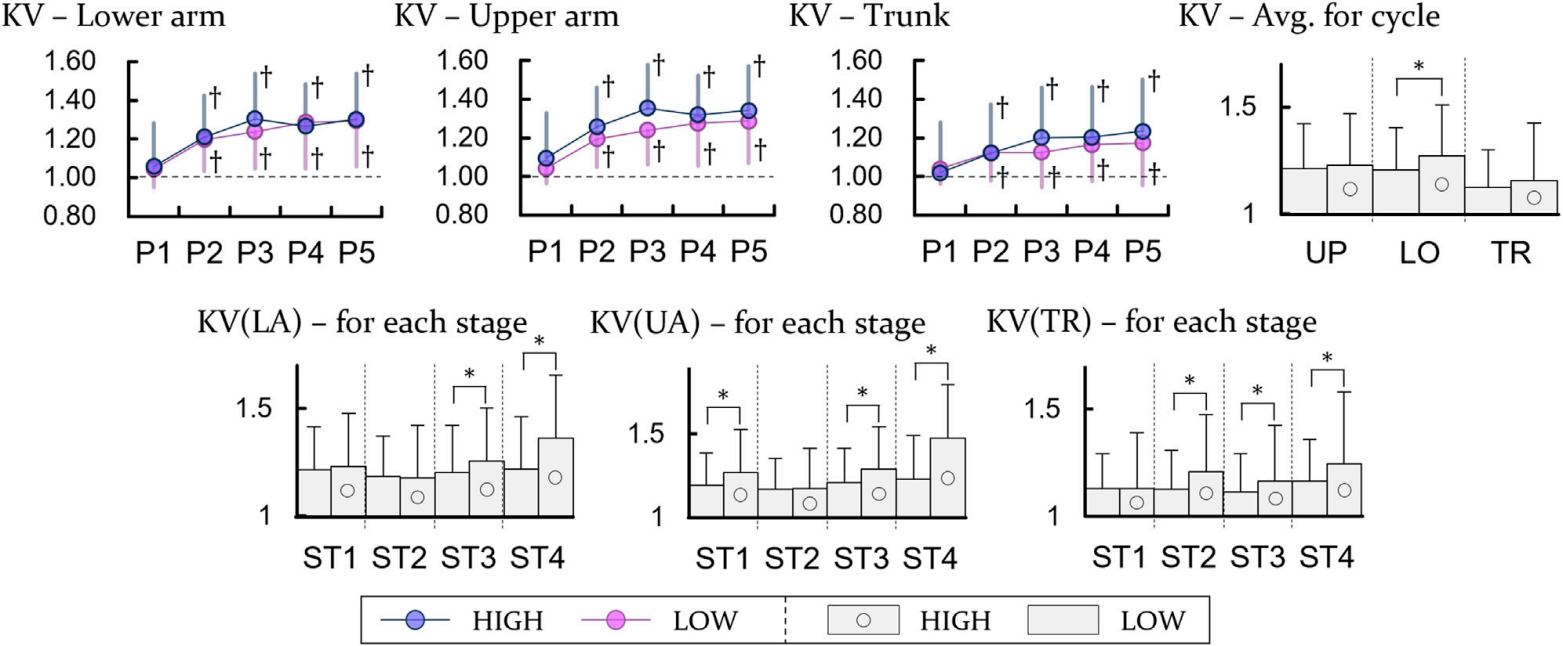
Experimental results of the KV. LA, UA, and TR are the lower, upper arm and trunk, respectively. P and ST indicate period and stage. The units of all variables are ratio to the result for the first bin of the LOW height. † represents a significant difference with regard to P1 (*p* < 0.0125). For comparing between the height, * means a significant difference with *p* < 0.05.

The cycle variation tended to increase in the lower arm, upper arm, and trunk as the task time elapsed and the number of periods increased (*p* < 0.0125). Additionally, in the case of the upper arm, the cycle variation increased as the height of the task increased, and no difference was found between the lower arm and trunk. However, in the variation results calculated by dividing a cycle into a total of four sections, one between each touch screen, some significant increases in cycle variation due to the increase in height were observed with the lower arm and trunk (*p* < 0.05) (Figure 4).

### 3.3 Movement stability

**FIGURE 5:**
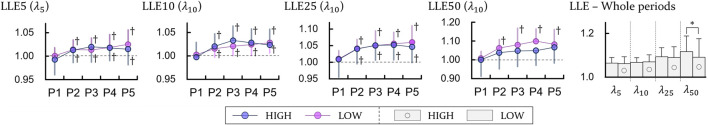
Experimental results of the local dynamic stability. P and ST indicate period and stage. The units of all variables are ratio to the result for the first bin of the LOW height. † represents a significant difference with regard to P1 (*p* < 0.0125). For comparing between the height, * means a significant difference with *p* < 0.05.

Three principal components extracted to calculate the local dynamic stability showed averages of 67%, 12%, and 7% influence for all participants; thus, they represented approximately 86% of the whole data. In the case of the calculated LLE, all four indicators increased over time (*p* < 0.0125) except for high height with LLE50, indicating that the subject’s movement stability decreased. A height comparison showed a significant difference only in the half-cycle stability ( $\lambda_{H}$ ; *p* < 0.05) (Figure 5).

### 3.4 Movement accuracy

**FIGURE 6:**
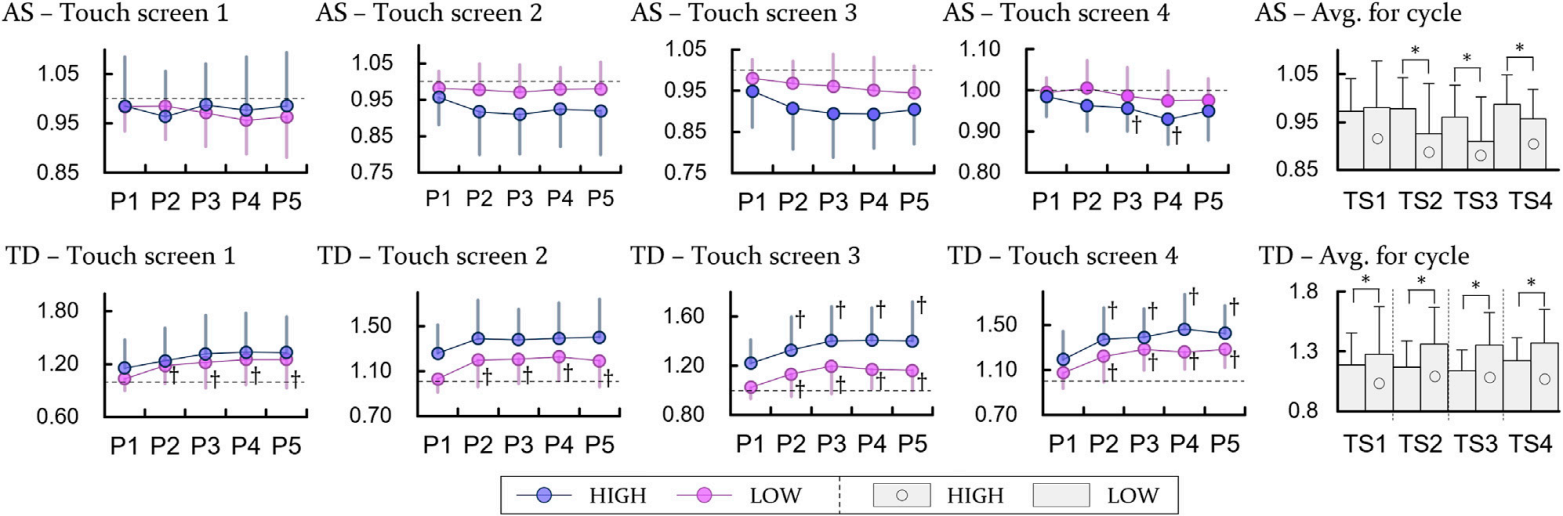
Experimental results of the task performance. AS and TD indicate the accuracy score and tapping deviation, respectively. P and ST specify period and stage. The units of all variables are ratio to the result for the first bin of the LOW height. † represents a significant difference with regard to P1 (*p* < 0.0125). For comparing between the height, * means a significant difference with *p* < 0.05.

For accuracy scores measured to assess movement accuracy, a decrease with time elapse was partially observed in TS4 of the HIGH height (*p* < 0.0125), and there was no significant comparison for the other cases. However, for the comparison in the context of the height difference, the accuracy scores of the HIGH heights had lower values than those of the LOW heights except TS1 (*p* < 0.05) (Figure 6).

In the case of the tapping deviation, all TSs showed an increase with time (*p* < 0.0125) except for high height with TS1 and 2. The significant difference according to the heights was shown for all sensors (*p* < 0.05), which indicates that the precision of the movement (tapping deviation) deteriorated with increasing table height.

### 3.5 Task time

**FIGURE 7:**
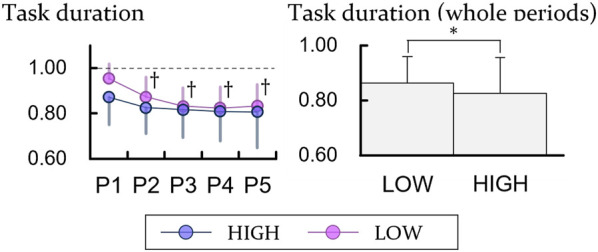
Experimental results of task duration. The units of all variables are ratio to the result for the first bin of the LOW height. P means period and † represents a significant difference with regard to P1 (*p* < 0.0125). For comparing between the height, * means a significant difference with *p* < 0.05.

The task time of the experiments with low height decreased with elapsed time, which shows that participants conducted the tasks progressively faster (*p* < 0.0125). In the comparison for the heights, they moved faster with the HIGH heights (*p* < 0.05) (Figure 7).

### 3.6 Simulation

**FIGURE 8:**
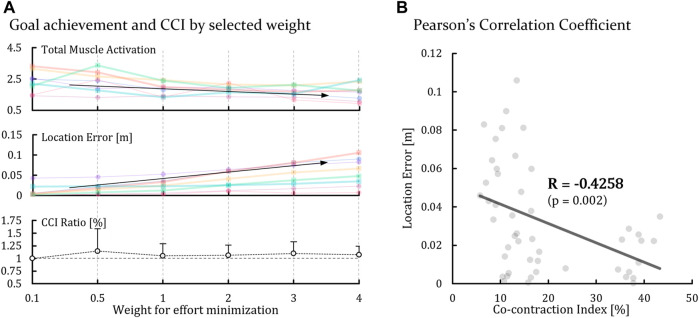
Results of the simulation using OpenSim. **(A)** Total muscle activation, location error and CCI ratio with several weights for effort minimization. **(B)** Correlation coefficient between the location error and cocontraction index.

The results of the predictive simulation using OpenSim show that the sum of the muscle efforts decreased and the pointing error increased according to the increase in the weights selected for muscle effort conservation. Errors for each period were calculated from the value by pointing to the TSs located at the end of the periods; for example, the error of P1 was from TS2. The pointing errors of ST1 and ST2 were higher than those of ST3 and ST4. In contrast, in the case of the CCI, which was calculated from the anterior and posterior muscle activations of the deltoid, ST3 and ST4 showed higher values than ST1 and ST2. The CCI had a moderate relationship of −0.4258 (*p* = 0.002) with the pointing error, which was calculated using Pearson’s correlation analysis and showed that pointing error decreased with increasing CCI (Figure 8).

## 4 Discussion

**FIGURE 9:**
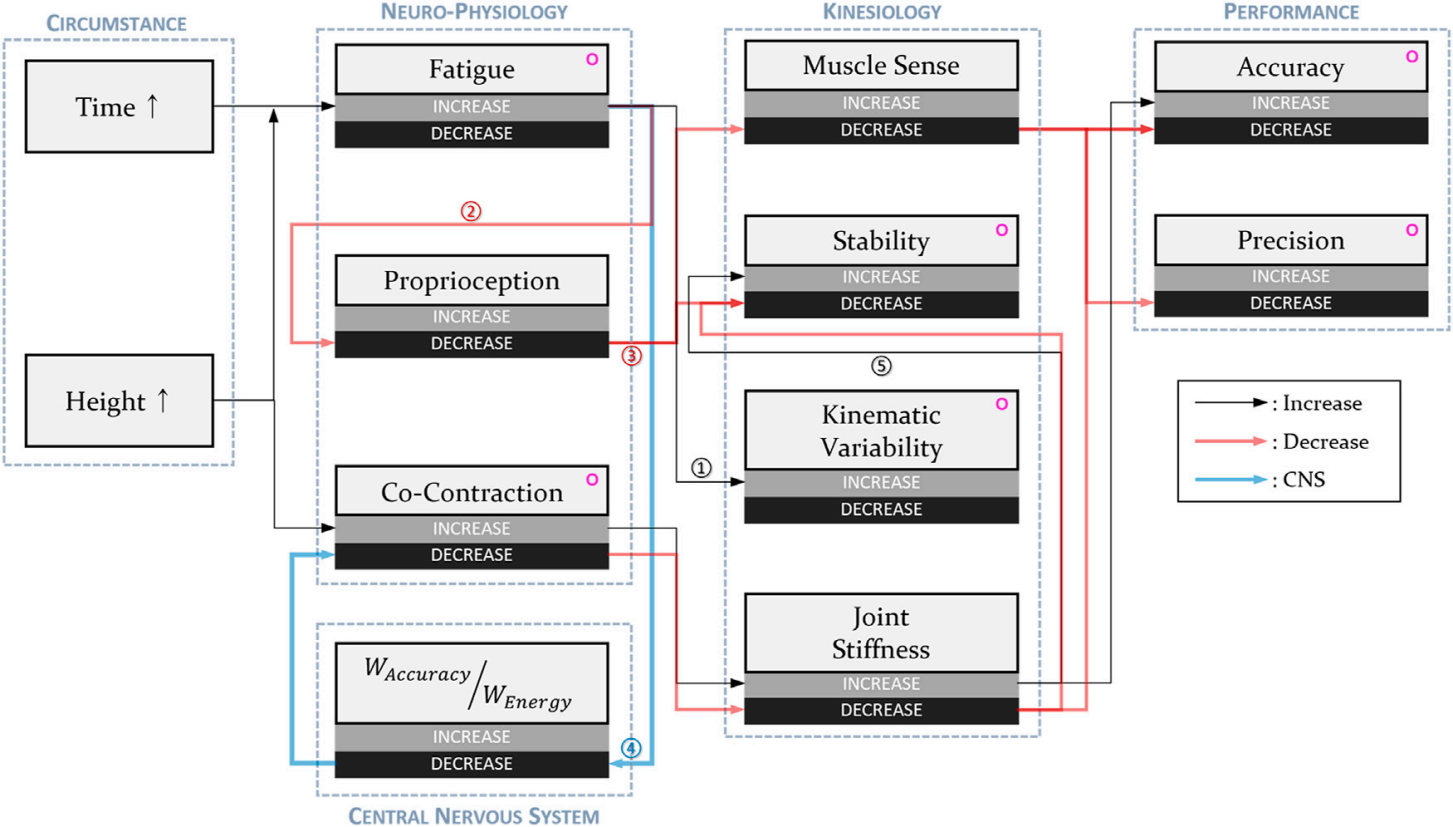
Blocks of all elements related to the human movement and addressed in this study, including the circumstances, neuro-physiological elements, kinesiological elements, and performances. Some arrows are numbered because they are used for explanation in the discussion. Circles with magenta color represent the indices that were measured in this study.

This paper explained how accumulated fatigue with elapsed time and different heights affect the task performance of the participants who conducted repetitive fine manipulative tasks with neuro-physiological and kinesiological elements. We assumed that as the task time elapsed and the height of the table increased, the amount of accumulated fatigue on the shoulder muscles would increase, and it would change the activation of the muscles and subsequent movement so that the task performance eventually deteriorated. To briefly explain the results, accumulated fatigue of the anterior and posterior deltoid actually increased with time and height, and the accuracy score decreased and tapping deviation increased for both cases. However, as shown in Figure 9, it cannot be easily concluded because there are many elements related to the end-point accuracy, such as proprioception, muscle cocontractions and several kinesiological elements.

### 4.1 Time elapsed

As the task duration increased with time, the median frequencies of the anterior deltoid tended to decrease gradually at both the LOW and HIGH heights (*p* > 0.0125). The median frequencies of each period for the medial deltoid at the HIGH heights showed a decreasing trend, but it was also not significant (*p* > 0.0125). Normally, when conducting a redundant manipulative task with the upper limb, if fatigue accumulates in a certain muscle, the CNS changes the movement strategy for dividing the load to other muscles to disperse fatigue (Arrow No. ① in Figure 9) (Prilutsky et al., 1997; Rasmussen et al., 2001). This strategy leads to an increase in cycle variation of repetitive motions (Kang and Dingwell, 2009; Gates and Dingwell, 2011; Longo et al., 2018a) and an increase in the kinematic variability for the three segments of the upper limb with the time elapsed regardless of the height difference, showing that common notions can also be applied to this experiment. We know from the results that time elapse of the task accumulated fatigue in the part of the shoulder muscles.

It is known that accumulated fatigue deteriorates proprioception, which leads to postural instability. Proprioception is used for postural equilibrium, joint stability, and control of the muscle sense so it is essential for achieving the purpose of a movement task with high performance. A specific pathway was not discovered regarding how muscle fatigue results in the deterioration in proprioception, but some research found through experiments that general fatigue load affects the proprioception of joints (Blasier et al., 1995; Voight et al., 1996; Abd-Elfattah et al., 2015). Fatigue can make muscle weak and deteriorate proprioception (Arrow No. ② in Figure 9), and proprioception can also deteriorate as the CNS changes efferent discharge patterns according to fatigue for energy efficiency if fatigue is generated in other muscles. If proprioception worsens in either way, subjects will have difficulty in controlling their limbs as precisely as they want, which worsens postural stability (Arrow No. ③ in Figure 9), and this was observed in our experiments with the results of the movement stability, which deteriorated with the time elapsed, as shown in Figure 5.

Gates and Dingwell (2011) performed experiments with human subjects using a sawing task with two different heights and analyzed the effects of accumulated fatigue on local dynamics stability, but they did not observe a difference in the context of height, which is different from our results. They guessed that subjects changed the movement pattern according to the accumulation of the fatigue so that they could minimize the deterioration of the stability by switching the muscles that were responsible for the movement, but they did not prove it with quantitative data. However, kinematic variability and local dynamic stability normally have a positive relationship, and it could be a reasonable thought that the effects of the initial perturbation on the deviation of the trajectory are much larger when the variation between the cycles increases. The difference between their experiments and ours is the characteristics of the imposed tasks. Their experiments were a dynamic sawing motion, which requires high muscular strength, and in contrast, ours was a fine manipulative task that requires high accuracy of the end point rather than muscular power. They said that their experiments also measured the task precision with a metronome for constant tempo, but it can be regarded as the accuracy of a manipulative task. In our experiments, subjects wanted to maintain the end-point accuracy for conducting the required task so that changing movement strategy was limited compared with their experiments, which makes it difficult to disperse the fatigue to other muscles and consequentially leads to the deterioration of the stability.

In fact, if a task requires higher end-point accuracy, muscles show larger fatigue. Hunter et al. (2002) performed human subject experiments to compare muscle activity during positioning and force tasks and found that endurance time until the end of the task with the subjects’ exhaustion was shorter with the positioning task, although the force task required more muscular power based on EMG activity. The increase in the mean arterial pressure, a rating of perceived exertion, the EMG of the anterior deltoid, and fluctuations in motor output were significantly larger with the positioning task. These results indicate that fatiguing occurred more with the positioning task so that the inhibitory input for the motor neuron increased, which led to a high exertion rate of the participants. Unlike the experiments of Gates et al., participants of our experiments were instructed to tap the center of the circle as much as possible so that the effects of the fatigue increased, and stability could be more deteriorated because of this.

Changes in task performance were also observed over time. The accuracy score and tapping deviation, which were measured to evaluate task performance, decreased with time. Previous studies found that muscle sense from proprioception and joint stiffness controlled by cocontraction affect the performance of positional tasks (Figure 9) (Abd-Elfattah et al., 2015). Normally, the CNS decreases the level of cocontractions regardless of how much energy is left for muscle activation to not be caught in a vicious cycle for metabolic efficiency when fatigue increases (Missenard et al., 2008). However, changes in the CCI were not observed as time elapsed, and we hypothesize that this is because participants had to maintain end-point precision for conducting tasks that have the characteristics of positioning.

Although it is not exactly the same as the real situation, the result of the predictive simulation showed a similar phenomenon to the results of the CCI (Figure 8A). Looking into it, we found that as the weight for minimizing the total muscle activation increased, total muscle activation decreased, and the final position error of the end point increased, which was the same as the basic principle of the CNS (Arrow No. ④ in Figure 9). However, when calculating the CCI using muscle activation of the anterior and posterior deltoids using the same method as in a previous study (MacIntosh and Keir, 2017; Shabani and Stavness, 2018), even if the size of the entire muscle activation was reduced, the CCI result did not change (Figure 8B). This is because even if the CNS increases the weight for energy conservation in response to fatigue, a certain level of cocontraction must be maintained to perform a positioning task, which means that fatigue cannot affect the level of cocontraction, which supports the claim that the CCI did not change with elapsed time.

Then, muscle sense from only proprioception remains, which can affect task performance. Although we did not quantitatively measure the muscle sense, it is clear that proprioception deteriorates due to fatigue, and this is also applied to the shoulder joint (Carpenter et al., 1998). Therefore, as the position sense deteriorated, it became difficult for the participants to accurately control the upper limb, and because of the decreased proprioception, it became difficult to tap where they wanted to tap, which resulted in a drop in the accuracy score, and it was also difficult to press the same place during repeated trials, which increased the tapping deviation. Contrary to our results, Emery and Cote’s experiment found that the shoulder joint could maintain the end position sense using the elbow and wrist joints, even though proprioception was deteriorated by fatigue (Emery and Cote, 2012). However, since the end position sense had a range of up to 10 cm, which they evaluated before and after fatigue, it cannot be said to be a task requiring high precision, and their experimental process also did not add difficulty to the task for the evaluation of proprioception. However, since the positioning task we added was a fine manipulative movement with a diameter of only approximately 3 cm, the deterioration of the end position sense could not be prevented despite the arm being a multi-joint system and the imposed task having redundant characteristics. Therefore, it can be inferred that as the task difficulty increases, the effect of shoulder fatigue on the sense of end position becomes larger.

### 4.2 Height of the workspace

Another condition we would like to discuss, the difference in height, also resulted in a difference in task performance. Fatigue and cocontraction are neuro-physiological elements that vary with height. Performing tasks at HIGH heights requires sustained shoulder abduction, which is supported by the experimental results that showed a significant increase in RMS levels in all three shoulder muscles at the HIGH heights compared with the LOW heights. In addition, this persistent muscle use led to fatigue, which was shown as a significant reduction in the median frequency more with the HIGH heights than the LOW heights (Figure 3). Therefore, the effect of fatigue on task performance will be greater at a HIGH height. As mentioned before, kinematic variability is related to fatigue, and the results of kinematic variability calculated based on the entire cycle of the upper arm showed a significant increase at a HIGH height because the upper arm is directly connected to the shoulder joint. Task performance also deteriorated with a decrease in accuracy score and an increase in tapping deviation, and it can be interpreted that it was because of the deterioration of proprioception.

Cocontraction also depends on the height, and in the case of the CCI, it was discovered that it significantly increased at HIGH heights. As cocontraction increased, joint stiffness also increased, which increased the impedance of the joint to cope well with external perturbations so that the movement was more stable (Arrow No. ⑤ in Figure 9). In the case of movement stability, the result of $\lambda_{H}$ calculated based on the half cycle showed that the movement of the participants was more stable at the HIGH height, proving that movement was stabilized when cocontraction increased. The experiment of Gates and Dingwell (2011) also showed the same phenomenon with stable movement despite increased fatigue and kinematic variability at the HIGH height, and they suggested that this was because of the increase in cocontraction, although they did not measure it. Based on the fact that the effects of cocontraction and fatigue on movement stability are contrary, the results of stabilized movement with HIGH height inferred that the effect of the cocontraction and subsequent joint stiffness have more impact on the movement stability than the increased fatigue.

However, the impacts of these two on task performance were reversed. Normally, an increase in joint stiffness accompanied an improvement in end-point accuracy, but it was difficult to find this trend in our experimental results, and task performance actually decreased with the increase in cocontraction at the HIGH height. This inferred that the effect of the muscle sense had more effect on the fine manipulative task than that of joint stiffness. Even if the movement stabilized with the increase in cocontraction due to the increase in height, proprioception was more deteriorated according to the increase in fatigue so that the end-point accuracy, which depends on the muscle sense, was also deteriorated and was not enough for conducting the fine manipulative task. Looking at the results of the predictive simulation using OpenSim, the error of the final location and the cocontraction between the anterior and posterior deltoids showed a moderate negative relationship, indicating that task performance should be improved with HIGH height with an increase in cocontraction (Figure 8A). However, we did not consider the effects of muscle fatigue and muscle sense, which depend on proprioception, for the simulations. The simulation algorithms calculate the optimized movement based on the weight for the muscle efficiency and final location error, similar to a simplification of the CNS process. If there is no generated fatigue, end-point accuracy should increase with those negative relationships and the increased cocontraction; however, deteriorated proprioception with accumulated fatigue, which was not considered in the simulation, led to the deterioration of the ability to control the motion precisely.

### 4.3 Task duration

The results of the task duration showed that participants conducted the task faster as time progressed or with higher height. This is aligned with the results of previous studies showing that participants tend to maintain discomfort from fatigue at a certain level (Rosa et al., 1998). Movement is more unstable when the speed of the movement is faster for repetitive motion (Asgari et al., 2015), and the cycle time of our experiments also decreased with the time elapsed regardless of height so that the movement stability deteriorated. Because of the increased fatigue, participants conducted the tasks faster at the HIGH heights. However, although the cycle time was shorter with the HIGH height, the stability of the movement was improved. We interpreted that this is because of the effects of the increased joint stiffness according to the cocontraction and again confirmed that joint stiffness has more impact on movement stability than fatigue, proprioception, or muscle sense.

## 5 Conclusion

We established two hypotheses prior to the experiment. We made equipment capable of performing fine manipulative tasks, recruited participants, and tried to verify them by collecting biomechanical data through several sensors. The first hypothesis is that the performance of fine manipulative tasks will be affected by inadequate table height. We discovered that the inappropriate height of the table caused fatigue in the deltoid and interrupted end point control, deteriorating the performance of tasks requiring high accuracy. And it was found that this phenomenon was strengthened as the height of the table increased and time continued. It can be concluded that proper height of the table is important in tasks that require high end point accuracy, and this is directly related to the task performance, so shoulder fatigue should be properly managed.

The second one was that there might be some relationship between end point accuracy and movement stability. We discovered that kinematic variability and largest Lyapunov exponent indicating movement stability increased as muscle fatigue accumulated, and task performance also deteriorated accordingly. We expected that movement stability and task performance had some relationship because performance would improve if the system was more stable. However, we could not determine if those two have a direct relationship and found that we cannot simply discuss the relationship because many elements are involved in fine manipulative tasks. Deterioration of end-point-related task performance due to accumulated fatigue cannot be fully compensated for by an increase in movement stability by controlling the joint stiffness with cocontraction. This leads to that the movement stability cannot be a sole index for evaluating the task performance of upper extremities which requires high precision, and we can tell that other elements such as work environment, neuro-physiological states, and ability for motor control with proprioception, should be considered simultaneously.

This study has a limitation that effect of learning was not considered in establishing the process of the experiments. Most of the participants (except 3 participants) did the experiment first with LOW height so that they might be able to learn the process of the experiment before another with HIGH height. However, it can be argued that this paper has a sufficient contribution because given task was not complex so that role of learning was not that important and, even in this situation, task performance tended to be more deteriorated at HIGH height. Therefore, the large flow of arguments in the discussion is not expected to be greatly affected.

## Data Availability

The raw data supporting the conclusion of this article will be made available by the authors, without undue reservation.
